# Early embryo development in a sequential versus single medium: a randomized study

**DOI:** 10.1186/1477-7827-8-83

**Published:** 2010-07-07

**Authors:** Goedele Paternot, Sophie Debrock, Thomas M D'Hooghe, Carl Spiessens

**Affiliations:** 1 Leuven University Fertility Centre, UZ Gasthuisberg, Campus Gasthuisberg, Herestraat 49, 3000 Leuven, Belgium

## Abstract

**Background:**

The success of in vitro fertilization techniques is defined by multiple factors including embryo culture conditions, related to the composition of the culture medium. In view of the lack of solid scientific data and in view of the current general belief that sequential media are superior to single media, the aim of this randomized study was to compare the embryo quality in two types of culture media.

**Methods:**

In this study, the embryo quality on day 3 was measured as primary outcome. In total, 147 patients younger than 36 years treated with IVF/ICSI during the first or second cycle were included in this study. Embryos were randomly cultured in a sequential (group A) or a single medium (group B) to compare the embryo quality on day 1, day 2 and day 3. The embryo quality was compared in both groups using a Chi-square test with a significance level of 0.05.

**Results:**

At day 1, the percentage of embryos with a cytoplasmic halo was higher in group B (46%) than in group A (32%). At day 2, number of blastomeres, degree of fragmentation and the percentage of unequally sized blastomeres were higher in group B than in group A. At day 3, a higher percentage of embryos had a higher number of blastomeres and unequally sized blastomeres in group B. The number of good quality embryos (GQE) was comparable in both groups. The embryo utilization rate was higher in group B (56%) compared to group A (49%).

**Conclusions:**

Although, no significant difference in the number of GQE was found in both media, the utilization rate was significantly higher when the embryos were cultured in the single medium compared to the sequential medium. The results of this study have a possible positive effect on the cumulative cryo-augmented pregnancy rate.

**Trial registration number:**

NCT01094314

## Background

The success of in vitro fertilization techniques is defined by multiple factors including embryo culture conditions, related to the composition of the culture medium [1-3]. Currently, there is discussion about the ideal composition of culture media with two opposing views "back to nature" and "embryo free choice".

According to the "back to nature/need for sequential medium" principle, embryo culture media mimic in vivo conditions when the zygote moves from the fallopian tube to the uterus during early development [4-7]. In order to fulfill the needs of the embryo at each moment in development and to optimize the embryo quality, the sequential medium contains a different composition during different days of culture [5]. These "ideal" compositions are based on studies on animal models reporting a change of energy requirements as the in vitro culture of pre-implantation embryos evolves over time [8-10]. Moreover, molecules like EDTA, glutamine and some amino acids have been reported to have a variable effect on the embryo during development [4,11-13].

According to the "embryo free choice/singe culture medium" paradigm, the embryo is cultured in a single medium which is constant and contains all the components needed during its development and the composition of this single medium does not change during embryo culture [8]. Proponents of this paradigm argue that there is no direct experimental evidence that sequential media are required for optimal embryo development [8].

It is not clear which type of culture media (sequential or single) is associated with the best quality of embryos on day 2, day 3 or day 5 or, more importantly, with the highest implantation rate per embryo transferred. No differences in embryo quality on day 3 and day 5 were found between a single medium and a sequential medium [14]. Optimal embryo quality has been reported after embryo culture in either a sequential [1,15] or a single medium [16-18]. However, the quality of these studies was limited due to lack of randomization [1,15], or, in case of randomization, due to the lack of a specific hypothesis with a power calculation [17,18] or the failure to include a sufficient number of embryos [16]. It is not clear how the clinical implantation rate per embryo transferred is affected by embryo culture since only a few studies evaluated the difference in implantation rate with no significant advantage using one type of medium [1,18]. The latter can be due to the fact that there are an insufficient number of embryos transferred.

In view of the lack of solid scientific data and in view of the hypothesis that sequential media are superior to single media, we carried out a prospective randomized study to evaluate the difference in embryo quality on day 1, day 2 and day 3 between a sequential media (Sydney IVF Cook, Queensland, Australia) and a single media (Gynemed, Lensahn, Germany).

## Methods

### Patient selection

A total of 178 patients, younger than 36 years, who entered their first or second IVF/ICSI cycle (each patient was included only once) between January 2008 and January 2009 at the Leuven University Fertility Centre, were informed about this prospective randomized study. The study was approved by the medical ethics committee of the University Hospitals Leuven (ML 4562). A total of 8 patients refused to participate. Patients were excluded when the cycle included biopsy for pre-implantation genetic diagnosis or if donor sperm/donor oocytes were used (table 1). A total of 170 patients were randomized, after informed consent, by a sealed envelope system. At random the embryos of the patient were cultured in a sequential medium (group A: Sydney IVF fertilization medium - Sydney IVF cleavage medium, Queensland, Australia) or in a single medium (group B: GM501 - GM 501, Gynemed, Lensahn, Germany) covered with mineral oil (Gynemed, Lensahn, Germany). In total, the dataset contained 85 patients in each group. Cycles with a failed fertilization (n = 7), an embryo transfer on day 2 (n = 13) or cryopreservation of all the fertilized oocytes on day 1 (n = 3) were eliminated (table 1). After this elimination step, group A contained 70 patients (419 embryos) and group B included 77 patients (583 embryos). In total, 28 patients entered their second cycle (group A: n = 12; group B: n = 16). An overview of patients and cycle characteristics are listed in table 2. The stimulation protocol used in our study is described in detail by Debrock et al. [19].

**Table 1:** Number of patients/cycles eligible, informed, excluded and included in the study

		GROUP A	GROUP B
**Number of patients eligible (n)**	358		
**Number of patients informed (n)**	178		
**Number of patients accepted (n)**	170		
**Response rate (%)**	96%		
**Number of patients randomized**	170	85	85
**Number of cycles excluded after randomization**			
**- Failed fertilization (n)**	7	4	3
**- Cryopreservation on day 1 (n)**	3	3	0
**- Embryo transfer on day 2 (n)**	13	8	5
**Number of cycles included for analysis (n)**	147	70	77

**Table 2:** Patients' and cycles characteristics of the study objects

	GROUP A (n = 70)	GROUP B (n = 77)
**Patient characteristics**		
Mean Age	30.0 (± 3.0)	29.9 (± 3.2)
Cause of subfertility		
*Tubal factor*	7 (10%)	7 (9%)
*Ovulation*	17 (24%)	14 (18%)
*Endometriosis*	16 (23%)	13 (17%)
*Implantation*	3 (4%)	4 (5%)
*Other*	6 (9%)	9 (12%)
*Male factor*	57 (81%)	50 (65%)

**Cycle characteristics**		
Number of first cycle patients (number of embryos)	58 (354)	61 (455)
Number of second cycle patients (number of embryos)	12 (74)	16 (128)
Number of ICSI cycles (%)	31 (44%)	32 (42%)
Number of IVF cycles (%)	39 (56%)	45 (58%)
Mean number of oocytes/PU (± SD)	11.1 (± 5.3)	11.3 (± 5.1)
Mean number of mature oocytes/PU (± SD)	9.6 (± 4.3)	10.2 (± 4.9)
Fertilization rate/oocyte (± SD)	54% (± 20)	67% (± 20%)
Fertilization rate/mature oocyte (± SD)	63% (± 21)	74% (± 17%)

### IVF/ICSI procedure

During oocyte retrieval, the oocytes of patients of group A were kept in Sydney IVF Gamete buffer and those of patients of group B were placed in GM 501 Wash. After oocyte retrieval, oocytes were washed through 4 wells each containing 500 μl fertilization medium (group A: Sydney IVF fertilization medium, group B: GM501 culture) (37°C, pH: 7.25-7.35) under mineral oil. Spermatozoa used for the IVF procedure were prepared using standard density gradient procedures (Isolate, Irvine Scientific, USA). Sperm samples used for ICSI, including TESE samples(group A: n = 7; group B: n = 5), cryo samples (group A:n = 1; group B: n = 1) and samples with a low motility and/or low concentration (group A:n = 23; group B: n = 26), were diluted and were centrifuged two times during 10 minutes (300 g). During sperm preparation Sydney IVF Gamete Buffer and Fertilization medium were used in group A versus GM501 Wash buffer and culture medium in group B. Standard in-vitro fertilization (IVF)/intracytoplasmic sperm injection (ICSI) procedures were performed 2-6 hours after oocyte retrieval. During the IVF procedure oocytes were inseminated with 200.000 progressively motile spermatozoa per well (0.5 ml). In case of an ICSI cycle, injected oocytes were incubated together in a 20 μl culture medium droplet under oil.

### Embryo quality assessment

On day 1, 16 h-20 h after insemination/injection, oocytes were examined for the presence of two pronuclei. All fertilized oocytes were rinsed and transferred to individual 20 μl droplets cleavage medium (group A: IVF cleavage medium; group B: GM501 culture) and incubated under mineral oil in order to follow their further individual development. The computer system (FertiMorph, Image House, Copenhagen, Denmark) was used to record image sequences of the embryos on day 1, day 2 and day 3 of their development (submitted by Paternot et al. 2010).

At day 1: the size and position of the pronuclei and the presence of a cytoplasmic halo were evaluated. A total of 25 embryos were excluded from this evaluation since visible pronuclei were not seen at the moment of image recording. Twenty-four hours after ICSI and twenty-six hours after IVF, a number of embryos (n = 471, 172 embryos from group A and 299 embryos from group B) were additionally evaluated on day 1 for the of early cleavage. This additional analysis was not performed during weekends.

At day 2 (41 h-44 h after insemination/injection) and day 3 (66 h-71 h post-insemination/injection) all the embryos were scored for the number and the size of blastomeres (equal or unequal (> 25% difference in size)), the degree of fragmentation (0 = no fragmentation, 1 < 10%, 2 = 10-25%, 3 = 26-50%, 4 > 50%) the size (small or large) and the position (local or dispersed) of the fragments. In addition, on day 2 the presence of multinucleation and the orientation of the cleavage axes (when a four cell was available) were evaluated. Perpendicularly orientated cleavage axes were defined as normal orientated cleavage axes. An embryo at day 3 with 7,8 or 9 equally sized blastomeres was defined as a good quality embryo on day 3. At day 5 the blastocyst stage was evaluated based on the presence of the inner cell mass, the trophectoderm layer, the blastocoel and the degree of expansion as described by Gardner and Schoolcraft [20]. A blastocyst with a blastocoel completely filling the embryo, a tightly packed inner cell mass and a trophectoderm with many cells forming a cohesive epithelium was defined as a good quality blastocyst on day 5.

### Embryo transfer and cryopreservation

After selection of the best embryo based on the morphological characteristics, embryo transfers were performed on day 3 or day 5. Regarding the Belgian legislation, single embryo transfers were performed when the patients entered for the first time an IVF/ICSI cycle. When they participated for the second time, the number of embryos transferred depended on the embryo quality. When a good quality embryo was available, a single embryo transfer was performed; in other cases two embryos were transferred. Embryos with at least 6 blastomeres, a degree of fragmentation less than 25% and equal sized blastomeres were cryopreserved on day 3. Embryos reaching the blastocyst stage (minimum expansion: early blastocyst; inner cell mass and trophectoderm layer: score A or B [20]) on day 5 were cryopreserved on day 5. Utilization rate was defined as the number of embryos available for cryopreservation and embryo transfer over the total number of embryos. Implantation was defined as the number of gestational sacs observed, divided by the number of embryos transferred [21].

### Statistics

The embryo quality on day 3 was measured as primary outcome. According to a power analysis, it was calculated that 395 embryos were needed in each group to detect a significant (reference value 35%) difference in embryo quality of 10%, at a statistical power of 0.80. The embryo quality was compared in both groups using a Chi-square test with a significance level of 0.05.

## Results

### Fertilization rate

In total, 1645 oocytes were retrieved after pick-up with a mean number of 11.11 (± 5.31) oocytes per oocyte retrieval in group A and 11.26 (± 5.14) in group B. A total of 669 (86%) mature oocytes in group A and 788 (91%) mature oocytes in group B were inseminated on the day of oocyte retrieval. The fertilization rate was significantly higher in group B (74% (583/788)) compared to group A (63% (419/669)) (p < 0.001) (table 2) which could be due to a lower number of mature oocytes in group A. As a result, our dataset contained a total of 1002 embryos(419 embryos in group A and 583 embryos in group B) available for analysis, exceeding the number required (n = 395) according the above mentioned power analysis.

### Embryo quality on day 1 (group A: n = 410; group B: n = 567)

A total of 25 embryos (group A: n = 9; group B: n = 16) were excluded for the evaluation of the characteristics on day 1 (as described in the Materials and Methods section). When compared to group A, embryos from group B had more embryos with a cytoplasmic halo (group B:46% (258/567) versus group A: 32% (132/410) (p < 0.0001)). Embryos from both groups were comparable for the other characteristics evaluated on day 1 (table 3).

**Table 3:** Morphological characteristics of the embryos evaluated on day 1 (Chi-square test)

Characteristics day 1	GROUP A (n = 410)	GROUP B (n = 567)	p-value
Position of the pronuclei (central)	363 (89%)	526 (93%)	NS
Size of the pronuclei (equal)	363 (89%)	498 (88%)	NS
Presence of a cytoplasmic halo (present)	132 (32%)	258 (46%)	< 0,0001
Early cleavage (present)	34 (20%)	59 (20%)	NS

### Embryo quality on day 2 (group A: n = 419; group B: n = 583)

There was a significant difference in the number of blastomeres, the degree of fragmentation and the size of blastomeres between both types of medium. The number of blastomeres was significantly higher in group B compared to group A (p = 0.0002). Significantly less embryos had 2 blastomeres (17% (100) versus 28% (119)) (p < 0.001) and significantly more embryos had at least 5 blastomeres (19% (110) versus 11% (45)) (p = 0.0004) on day 2 in group B (table 4).

**Table 4:** Morphological characteristics of the embryos on day 2 cultured in a sequential medium (group A) versus a single medium (group B) (Chi-square test)

Characteristics day 2	GROUP A (n = 419)	GROUP B (n = 583)	p-value
Number of blastomeres			
1	30 (7%)	24 (4%)	0.0353
2	119 (28%)	100 (17%)	< 0.0001
3	61 (15%)	102 (17%)	NS
4	164 (39%)	247 (42%)	NS
≥ 5	45 (11%)	110 (19%)	0.0004
Degree of fragmentation			
0	84 (20%)	140 (24%)	NS
1	91 (22%)	154 (26%)	NS
2	132 (32%)	158 (27%)	NS
3	90 (21%)	115 (20%)	NS
4	22 (5%)	16 (3%)	0.0405
Size of blastomeres (equal)	270 (64%)	294 (50%)	< 0.0001
Position of fragments (local)	237 (70%)	329 (74%)	NS
Size of fragments (small)	235 (69%)	326 (73%)	NS
Multinucleation (present)	22(6%)	42 (7%)	NS
Cleavage axes (normal)	125 (76%)	186 (75%)	NS

In addition, more embryos had a degree of fragmentation higher than 50% (score 4) in group A (5% (22)) versus group B (3% (16)) (p = 0.0405). Finally, embryos of group A had significant more equally sized blastomeres (64% (270)) compared to the embryos of group B (50% (294)) (p < 0,0001) (table 4). No differences in both groups were seen in the evaluation of the position and the size of fragments, the presence of multinucleation or the orientation of the cleavage axes (table 4).

### Embryo quality on day 3 (group A: n = 419; group B: n = 583)

The number of blastomeres of the embryos of group B was significantly higher compared to the embryos of group A (p < 0.0001). More embryos of group A (40% (169)) were evaluated with equally sized blastomeres compared to group B (30% (177)) (p = 0.0011) (table 5). For the other characteristics evaluated on day 3 no significant differences were found (table 5).

**Table 5:** Morphological characteristics of day 3 embryos cultured in both types of culture media (Chi-square test)

Characteristics day 3	GROUP A (n = 419)	GROUP B (n = 583)	p-value
Number of blastomeres			
≤ 5	203 (48%)	181 (31%)	< 0.0001
6	57 (14%)	97 (17%)	NS
7	67 (16%)	126 (22%)	0.026
8	73 (17%)	129 (22%)	NS
9	14 (3%)	32 (5%)	NS
≥ 10	5 (1%)	18 (3%)	0.0483
Degree of fragmentation			
0	52 (12%)	109 (19%)	0.0075
1	93 (22%)	130 (22%)	NS
2	141 (34%)	177 (30%)	NS
3	105 (25%)	137 (23%)	NS
4	28 (7%)	30 (5%)	NS
Size of blastomeres (equal)	169 (40%)	177 (30%)	0.0011
Position of fragments (local)	130 (40%)	179 (42%)	NS
Size of fragments (small)	219 (68%)	294 (68%)	NS
Good quality embryo	372 (89%)	526 (90%)	NS
Utilization rate	206 (49%)	326 (56%)	0.0346

### Embryo quality on day 5 (group A: n = 49; group B: n = 71)

No differences were found in the number of good quality blastocysts on day 5 between both groups (group A: 6% (3/49) versus group B: 6% (4/71)) (p = 0.1476).

### Utilization rate

Finally, the utilization rate was significantly higher in group B compared to group A (group B: 56% (326) versus group A: 49%(206)) (p = 0.0346) (table 5).

### Implantation rate

No difference was found between the implantation rate of an embryo in the first (29% (33/113)) or second IVF/ICSI cycle (41% (14/34)) (p = 0.3572). Five patients had no embryo transfer due to a high risk of OHSS (Ovarium Hyper Stimulation Syndrome) (2 patients of group A; 3 patients of group B). The embryos of these patients were cryopreserved on day 3. In group A, 3 double embryo transfers were performed on day 3, 60 single embryos transfers on day 3 and 5 single blastocyst transfers on day 5. In group B, 2 double embryo transfers on day 3, 65 single embryo transfers on day 3 and 7 single blastocyst transfers on day 5 were performed. In total, 147 embryos were transferred (71 embryos in group A, 76 embryos in group B). The implantation rate was significantly higher in group B (group A 20% (14/71) (day 3 2 ET: n = 4/6; SET day 3: n = 9/60; SET day 5: n = 1/5)), group B 28% (21/76) (day 3 2 ET: n = 0/4; SET day 3: n = 18/65; SET day 5: n = 3/7)) (p = 0.032).

## Discussion

This study showed for the first time that on day 3 the number of good quality embryos was comparable but the utilisation rate was higher after culture in a single medium than after culture in a sequential medium. This is the first randomized study that evaluated a unique set of characteristics separately on day 1, day 2 and day 3 of embryo development.

When compared to embryos cultured in sequential medium, embryos cultured in single medium were observed to have more frequently a cytoplasmic halo on day 1. A cytoplasmic halo is a manifestation of a microtubule-mediated withdrawal of mitochondria and other cytoplasmic components to the perinuclear region. The presence of a cytoplasmic halo has been reported to positively correlate with embryo quality and pregnancy rates [22-24]. This could not be confirmed by our data since the embryo quality was comparable on day 3 among halo positive embryos and halo negative embryos. In literature, the presence of an early cleavage is shown to correlate with embryo quality [25,26]. In our study no difference was found in the presence of an early cleavage between both types of culture media, possibly because our study was not powered to detect the difference in early cleavage rate between both groups or because such a difference does not exist.

When compared to embryos cultured in sequential medium, embryos cultured in single medium were observed to have a higher number of blastomeres on day 2 and day 3, suggesting that embryos develop faster in single medium than in sequential medium. This observation is in line with results reported by other investigators [17] in a different patient population (oocyte donors, mean age of recipients 40.06 ± 5.4 years). Ben-Yosef et al. compared two sequential media and reported a lower cleavage rate when embryos were cultured in the COOK IVF medium [27].

It is difficult to compare our results with those of other randomized studies with a similar design and aim [16,18] because they were either underpowered [16] or power was not calculated [18]. Furthermore, a combination of embryo morphological characteristics was used in the embryo scoring system of these studies [16,18] making it impossible to evaluate individual embryo characteristics. Nevertheless, both studies [16,18] reported, like our study, no differences in the number of good quality embryos on day 3. Biggers and co-workers [14] reported also individual embryo characteristics and found no differences in the number of blastomeres and the degree of fragmentation on day 3 when embryos were cultured in a single or sequential medium.

Although no significant difference could be found in the number of good quality embryos on day 3, in our study the utilization rate was significantly higher for the embryos cultured in the single medium. The study of Macklon et al. [16] reported a similar characteristic on day 5, namely the blastulation rate, which was equal for both types of culture media.

In our study, no statistically solid conclusions can be made on the level of embryo development on day 5 nor on the level of implantation since our study was not powered to compare these outcome variables between both groups. Some studies [14] found no differences in the blastocyst development on day 5 after embryos were cultured in a sequential or single medium while others found a benefit when the embryos were cultured in a single medium [17]. Other studies showed no significant difference in the implantation rate of embryos cultured in a single or sequential medium [1,16-18].

The fertilization rate was significantly higher in the single medium. This could be a possible explanation for the difference in embryo quality. Although in our study we cannot discriminate for the oocyte quality since this was not the scope of this study and therefore not included in the study design. Our observation that the embryo quality/embryo utilization rate was better after culture in single medium than in sequential medium lends some support to the "embryo free choice hypothesis and is in line with the conclusions from a recent review paper [8] failing to find strong evidence in the literature to promote the use of a sequential medium protocol based on biological reasons and proposing new studies to evaluate the impact of culture medium on the gene expression of the embryo.

In our study design, we did not compare embryo quality in different media using sibling oocytes, allowing each patient to serve as her own control. However, using sibling oocytes at the moment of fertilisation control can give a methodological problem. This is due to the fact that different types of culture media differ in osmolarity. Using sibling oocytes will result in extra stress to the embryos when the culture medium is changed after fertilization [14]. In this way, at the end of the study, the effect of the differences in osmolarity between available commercial media can also have an influence on the embryo quality which makes it difficult to make conclusions. However, using sibling oocytes, no differences were observed in embryo quality between single or sequential media in a previous paper [18].

## Conclusion

In conclusion, this is the first randomized study comparing a unique set of embryo morphological characteristics on day 1, day 2 and day 3 between a sequential and a single medium. On day 3 the number of good quality embryos was comparable but the utilisation rate was higher after culture in a single medium than after culture in a sequential medium, with a possible positive effect on the cumulative cryo-augmented pregnancy rate. Additional randomized trials are needed to test the hypothesis that these data can be confirmed for embryos cultured up till day 5.

## Competing interests

The authors declare that they have no competing interests.

## Authors' contributions

GP and CS contribute to the paper by defining the design of the of the study, the analysis and the interpretation of the data. Both authors draft the paper and approved the final version. SD and TD interpreted the data and revised the paper critically for important intellectual content and approved the final version. All authors read and approved the final manuscript
